# Community pharmacist intervention in depressed primary care patients (PRODEFAR study): randomized controlled trial protocol

**DOI:** 10.1186/1471-2458-9-284

**Published:** 2009-08-05

**Authors:** Maria Rubio-Valera, Antoni Serrano-Blanco, Pere Travé, M Teresa Peñarrubia-María, Mar Ruiz, Marian March Pujol

**Affiliations:** 1 Sant Joan de Déu – Serveis de Salut Mental, Fundació Sant Joan de Déu, Sant Boi de Llobregat, Barcelona, Spain; 2 Red de Investigación en Actividades Preventivas y Promoción de la Salud en Atención Primaria RedIAPP (RD06/0018/0017), Spain; 3 Estades en Pràctiques Tutelades Unit, School of Pharmacy, University of Barcelona, Barcelona, Spain; 4 ABS Just Oliveres, DAP L'Hospitalet, Àmbit Costa de Ponent, Institut Català de la Salut, L'Hospitalet, Spain; 5 ABS Bartomeu Fabrés Anglada, DAP Baix Llobregat Litoral, Àmbit Costa de Ponent, Institut Català de la Salut, Gavà, Spain

## Abstract

**Background:**

Treatment of depression, the most prevalent and costly mental disorder, needs to be improved. Non-concordance with clinical guidelines and non-adherence can limit the efficacy of pharmacological treatment of depression. Through pharmaceutical care, pharmacists can improve patients' compliance and wellbeing. The aim of this study is to evaluate the effectiveness and cost-effectiveness of a community pharmacist intervention developed to improve adherence and outcomes of primary care patients with depression.

**Methods/design:**

A randomized controlled trial, with 6-month follow-up, comparing patients receiving a pharmaceutical care support programme in primary care with patients receiving usual care. The total sample comprises 194 patients (aged between 18 and 75) diagnosed with depressive disorder in a primary care health centre in the province of Barcelona (Spain). Subjects will be asked for written informed consent in order to participate in the study. Diagnosis will be confirmed using the SCID-I. The intervention consists of an educational programme focused on improving knowledge about medication, making patients aware of the importance of compliance, reducing stigma, reassuring patients about side-effects and stressing the importance of carrying out general practitioners' advice. Measurements will take place at baseline, and after 3 and 6 months. Main outcome measure is compliance with antidepressants. Secondary outcomes include; clinical severity of depression (PHQ-9), anxiety (STAI-S), health-related quality of life (EuroQol-5D), satisfaction with the treatment received, side-effects, chronic physical conditions and socio-demographics. The use of healthcare and social care services will be assessed with an adapted version of the Client Service Receipt Inventory (CSRI).

**Discussion:**

This trial will provide valuable information for health professionals and policy makers on the effectiveness and cost-effectiveness of a pharmaceutical intervention programme in the context of primary care.

**Trial registration:**

NCT00794196

## Background

One of the main challenges of public health is to improve the treatment of depression. In fact, major depressive episode is one of the most prevalent mental disorders, both in the general population [1-3] and in primary care [4], and is one of the five mental disorders that cause the highest impairment, even higher than the ones associated with chronic physical conditions [5-7]. Furthermore, it is the most costly brain disorder in Europe, accounting for 33% of the total cost [8]. Even though pharmacological treatment in major depressive episodes is mostly prescribed as recommended by Spanish primary care physicians, percentages of treatment concordance with clinical guidelines are low (between 21% and 25%) mainly because recommended follow-up sessions are not performed [9]. Moreover, adherence to antidepressant medication is poor [10], which could limit its effectiveness in clinical practice. The World Health Organization and the European Council have stressed the importance of including community pharmacists, considered the health professional most readily accessible to patients, as an active member of the multidisciplinary healthcare team with the aim of benefiting patients' health [11,12], including those suffering from mental disorders [13].

By means of pharmaceutical care, community pharmacists have been shown to improve patient wellbeing in chronic physical conditions such as diabetes mellitus [14] and hypertension [15].

Research has been done to evaluate the effect of pharmaceutical care among outpatients diagnosed with depression [16-21] but in only three of the studies was the intervention conducted by a community pharmacist [16-18] and only one of them took place in a European country [16]. This was the only study that reported cost analysis information [22]. The results provided by these studies are contradictory and still more research is needed in order to study this issue. The aim of this study is to evaluate the efficacy of a pharmaceutical care programme, compared with usual care, on the improvement of adherence to antidepressant drugs and patient wellbeing in a population with a diagnosis of depression treated in primary care under real practice circumstances. Programme cost-effectiveness will be also calculated.

## Methods/design

We followed the CONSORT statement for reporting randomized trials [23].

**Figure 1:**
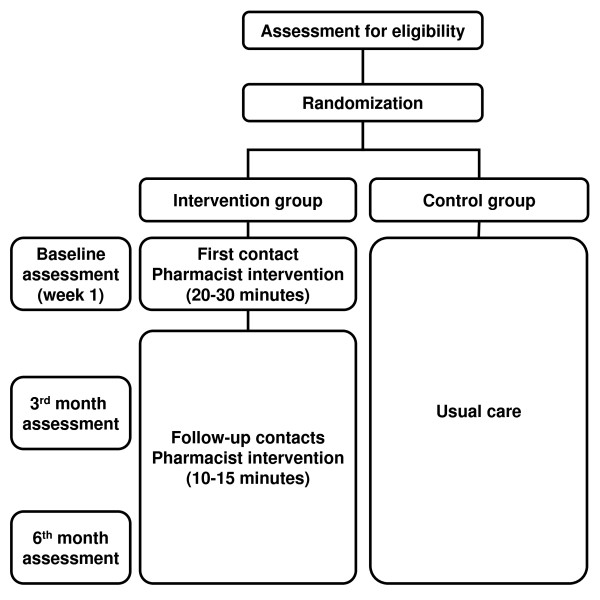
**Study design**.

6 month follow-up naturalistic randomized controlled trial with random allocation of participants into two alternative branches: 1) Usual medical and pharmaceutical care plus support programme in community pharmacy (intervention group), and 2) Usual medical and pharmaceutical care (control group) (Figure 1).

The evaluation of compliance and clinical improvement of participants will be carried out by individual assessment at baseline, 3 and 6 months after the beginning of the intervention.

The Clinical Research Ethics Committee of the Foundation Sant Joan de Déu (CEIC *Fundación *SJD) approved the study protocol (Reference Number: BECAFIS04/07). Participants are only allowed to enter the study after signed informed consent has been obtained.

### Setting

Gavà is a city situated in the province of Barcelona (Catalonia, Spain) approximately 15 kilometres south of Barcelona city. With a total area of 30.9 square kilometres and a population of more than 45,000 inhabitants, the city has two primary care health centres (PCHCs) (Gavà-1 and Gavà-Doctor Bartomeu Fabrés Anglada) that provide medical care to the whole population of Gavà. Patients will be recruited at those PCHCs from October 2008 to October 2009. 23 general practitioners (GPs) from the PCHCs voluntarily participate in the study and deal with the identification and subsequent recruitment of the patients.

Altogether there are 14 community pharmacies in Gavà that were asked to participate in the study. In addition, there is a community pharmacy in the adjacent town of Viladecans located very near to one of the PCHCs that was also asked to participate. Two of the pharmacies (13%) refused to participate, one citing heavy workload and the other a lack of interest in the study. Finally, 13 pharmacies with a total of 34 pharmacists will be responsible for providing patients with the intervention and usual care during the 6 month follow-up period.

### Enrolment, randomization and allocation

All patients initiating a treatment with any antidepressant due to a depressive disorder through medical prescription from a PCHC GP in Gavà, and who are aged 18–75, are candidates for inclusion in the study. The following patients will be excluded: those on antidepressant medication in the past 2 months, those who had an appointment with an specialist in mental health in the past 2 months, those with history of psychotic or bipolar disorders, those with history of drug abuse or dependency, those with cognitive impairment that prevents assessment interviews, and those attending a pharmacy not included in the study. The eligibility criteria are listed in Table 1.

**Table 1:** Eligibility criteria

**Patients...**
aged between 18 and 75,
initiating a pharmaceutical antidepressant treatment due to a depressive disorder through a medical prescription from a GP,
going to one of the participant community pharmacies,
that did not take antidepressant medication in the previous 2 months,
that have not had an appointment with an specialist in mental disorders in the previous 2 months,
with no history of psychotic or bipolar disorders,
with no history of drug abuse or dependency,
with no cognitive impairment that prevents assessment.

Patients meeting the inclusion criteria are given the information about the study's aim and procedures during the medical visit and written informed consent is obtained. With the informed consent, the GP registers the patient's telephone number and PCHC clinical-history reference number. Within a week of the inclusion date, baseline assessment is performed at the PCHC by a trained psychologist.

Randomization was generated at the patient level by a computerized random-number generator following a permuted block design. Block size was of 10 patients with a ratio of 1:1. To assure the concealment of allocation, every GP receives a set of 10 sequentially numbered, opaque, sealed envelopes containing patient assignment. Envelopes were generated by an external investigator and details of the series are unknown to any of the GPs or pharmacists in the study. As patients are enrolled, the GP sequentially staples one of the envelopes to the prescription. When the patient gives the prescription to their community pharmacist, they open the envelope and create a patient study chart distinguishing between control and intervention group.

Blinding of participants and pharmacists is not possible because of the type of intervention. However, the assessment visits and data analysis are conducted by independent and blinded evaluators.

### Intervention and usual care

Patients in the intervention group will receive the support programme in community pharmacy (PRODEFAR) every time they go to the pharmacy to pick up the medication or to ask for counselling in the course of the 6 months of the study. Pharmacists participating in the study received 8 hours of training about PRODEFAR prior to the study. The training followed a manual created for the study and is accredited by the Catalonian council of continuous pharmaceutical training (*Consell Català de la formació farmacèutica contínua*) (Reference Number: 09F00676).

PRODEFAR consists of a series of educational interventions focused on improving patients' knowledge of antidepressant medication, as well as making patients aware of the importance of compliance to the medication. Moreover, in patients with a sceptical attitude towards the medication, the intervention will aim to reduce stigma, reassure the patient about possible side-effects, and stress the importance of carrying out GPs' advice. First contact in the PRODEFAR is expected to take between 20 and 30 minutes, subsequent interventions are expected to take between 10 and 15 minutes.

Regardless of whether participants belong to the intervention group or not, they receive the usual pharmaceutical care as well as the treatment considered most appropriate by their physician. Patients receiving usual care get ordinary advice about medication when collecting it. Any concerns and questions addressed to the pharmacist are also answered.

Patients in the intervention group are asked to avoid conversations concerning the PRODEFAR with patients from the control group. The importance of this requirement is emphasised to patients from both groups at baseline assessment.

### Measurements

Three assessment visits – at baseline, 3 and 6 months – are conducted by independent and blinded interviewers. Participants, pharmacists and GPs are not blinded. To limit bias, two trained psychologists conduct all the interviews. ASB and MRV were responsible for the interviewers' training. Table 2 shows the measures taken at each assessment study visit.

**Table 2:** Measurement scheme

	**Instrument**	**T0**	**T1**	**T2**
**Baseline measures**				

Socio-demographics	Questionnaire	X		
Psychiatric diagnosis	SCID-I	X		
Chronic physical conditions	Check list	X		

**Effect evaluation**				

Compliance	Medication intake percentage	Continuous registration		
Compliance	MAQ		X	X
Severity of depression	PHQ-9	X	X	X
Health-related quality of life	EuroQOL-5D	X	X	X
Anxiety (state)	STAI-S	X	X	X
Side-effects	Check-list		X	X
Satisfaction	Armando PD questionnaire		X	X

**Economic evaluation**				

Direct and indirect costs	CSRI – adapted	X	X	X

T0 = Baseline, T1 = 3 months after baseline, T2 = 6 months after baseline.

SCID-I: Structured Clinical Interview Axis I DSM-IV; MAQ: Medication Adherence questionnaire; PHQ-9: Patient Health Questionnaire 9-item depression module; EuroQOL-5D: European Quality of Life Scale – 5 domains; STAI-S: State-Trait Anxiety Inventory (State subscale); CSRI: Client Service Receipt Inventory.

Clinical diagnosis is made using the research version of the Structured Clinical Interview for DSM-IV Axis I Disorders (SCID-I) [24,25]. Patients are interviewed in the modules of major depression (present and past), dysthymic disorder, anxiety disorder and adjustment disorder as defined according to DSM-IV criteria. Due to the pragmatic character of the study, GPs are blind to the DSM-IV diagnosis and patient inclusion and follow-up is performed according to their usual practice.

The primary outcome measure of our study is adherence to prescribed antidepressant medications, which is assessed through two methods:

1) pharmacy records: every time a patient buys their medication, the pharmacist registers the date of prescription, the date of dispensation and the number of pills dispensed. At 3 and 6 months from baseline, the patient is asked to present the stock of antidepressant medication and any surplus antidepressants following a GP recommended change in medication. To minimize bias, the patient is not told that the aim is to assess compliance. The pharmacist registers the stock of every medication and the percentage of medication intake is calculated by formula: (Number of doses removed/Number of doses prescribed)*100. Poor adherence is defined as taking less than 80% of the prescribed doses. There are two disadvantages of measuring adherence this way: the patient can remove pills but not take them, and this formula does provide information about the timing of the dose removal.

2) self-reported: adherence to prescribed antidepressant medications is assessed with the 4-item scale developed by Morisky et al [26]. The scale asks patients to respond "yes" or "no" to a set of 4 questions. A positive response to any question indicates a problem with adherence. Patients who respond "yes" to any of the items are categorized as non-adherent.

Clinical severity of depression is measured with the Patient Health Questionnaire 9-item depression module (PHQ-9) [27-29]. The PHQ-9 is a nine-item scale that assesses the depression symptoms of the Diagnostic and Statistical Manual of Mental Disorders (DSM-IV). Each of the nine items is scored from 0, not at all, to 3, nearly every day. The PHQ-9 can be used as a screening tool, with summed score ranging from 0 (no depressive symptoms) to 27 (all symptoms occurring daily). Summed scores of 0 to 4 correspond to minimal symptoms; 5 to 9 to mild symptoms; 10 to 14 to moderate symptoms, 15 to19 to moderately severe; and 20 to 27 to severe symptoms.

As mental disorders have been shown to be frequently comorbid in the general population in Spain, and the association between major depression and anxiety has been especially highlighted [4,30], it is recommended that comorbidity be taken into account when treating mental disorders. The State-Trait Anxiety Inventory (STAI) [31,32] is a 40 item self-report measure of state and trait anxiety. Total scores on the state subscale (STAI-S) (20 items) range between 0 and 60, with the higher scores indicating more severe state anxiety. STAI-S is administered in the present research to monitor comorbidity with depression and anxiety clinical severity.

Health-related quality of life is evaluated using the Spanish version of the EuroQol-5D (EQ-5D) [33-35]. The EQ-5D questionnaire is a generic instrument of health-related quality-of-life. The first of the two parts records self-reported problems in one of five domains-mobility, self-care, usual activities, pain/discomfort and anxiety/depression-divided into three levels of severity corresponding to; no problems, some problems, and extreme problems, thus generating 245 possible health states [36]. Each state corresponds to a single index value referred to as the tariff. Value 1.000 is the best health state and value 0.000 corresponds to being dead, 82 of the 245 states have negative values, and are thus rated as being worse than dead [36]. The second part records the subject's self-assessed health on a Visual Analogue Scale (VAS); a vertical 20 cm line on which the best and worst imaginable health states score 100 and 0, respectively.

Satisfaction with the treatment received from the pharmacist is measured with the patient satisfaction questionnaire developed by Armando PD et al [37]. This instrument consists of 10 closed questions using a 5-point Likert scale from 1 (total disagreement) to 5 (strong agreement) and an open section to express comments.

Evident side-effects are assessed using a brief version of the UKU [38] considering the most common side-effects of antidepressants. Those side-effects are listed in Table 3. For each side-effect the intensity (Not present, mild, moderate, or severe), frequency (high or low) and causal relation with antidepressant drugs (yes, no, or unclear) is assessed.

**Table 3:** Assessed side-effects

Asthenia/Lassitude/lncreased Fatigability
Sleepiness/Sedation
Tension/lnner Unrest
Increased Duration of Sleep
Reduced Duration of Sleep
Increased Dream Activity
Tremor
Increased Salivation
Reduced Salivation
Nausea/Vomiting
Diarrhoea
Constipation
Stomach cramp
Orthostatic Dizziness
Palpitations/Tachycardia
Headache
Increased Tendency to Sweating
Weight gain
Weight loss
Diminished Sexual Desire
Sexual dysfunction

Chronic physical conditions are assessed using a "yes" or "no" check-list of 28 illnesses with the potential to become chronic, and an open section for additional illnesses not considered by the authors of the list [39]. Physical conditions considered chronic are listed in Table 4.

**Table 4:** Assessed chronic physical conditions

Chronic Allergy
Arthritis/Rheumatism
Bronchitis/Emphysema
Asthma
Diabetes
Migraine/Chronic headaches
Chronic Back Pain.
Chronic Neck Pain
Vascular Diseases
Heart attack/Angina Pectoris
Heart diseases
Stroke
Varicose veins
Hypertension
Peptic or Duodenal Ulcer
Hemorrhoids
Chronic constipation
Psychological problems/Depression
Cataract
Vision impairment
Hearing impairment
Thyroid Diseases (Hiperthyroidism/Hypothyroidism)
Nervous System Diseases (Multiple Sclerosis, Parkinsonian Disorders, Epilepsy...)
Cancer
Acquired Immunodeficiency Syndrome/HIV Infection
(If male) Prostatic Diseases
(If female) Menopause

Additionally, patients are asked for socio-demographic details including age, sex, marital status, living arrangements, education, employment and type of contract.

### Economic evaluation

Client Service Receipt Inventory (CSRI) [40] is a questionnaire for collecting information about use of healthcare and social care services as well as other economic impacts. In our study, we adapted it to take into account the costs due to lost production as well as the cost of medicines, the costs of healthcare use and social care services, and the costs for patient in terms of travelling expenses and time lost. Patients are asked to give details of services and medicines that they have used during the previous 3 months due to depression or for other reasons. Services included hospital care, primary healthcare and social care, as well as the provision of aids, tests and medication. The length of stay is recorded for inpatient episodes, whilst the number of contacts with other services is recorded.

### Sample size

To calculate the sample size it was taken into account that we needed to obtain a difference of at least 17 points in the percentage of medication intake [16]. A total of 194 patients are needed to conduct the study, assuming an alpha risk of 0.05 and a beta risk of < 0.20 and a 20% dropout rate.

### Statistic analysis

Data collected will be analyzed using SPSS-WIN 17.0 and SAS 8.0 statistical analysis software and employs both quantitative and qualitative techniques. Firstly, comparability between the intervention and usual-care groups will be assessed at baseline to check differences.

### Effect evaluation

Data will be primarily analyzed according to the intention to treat principle (ITT), including all participants with valid data regardless of whether they did or did not receive the intervention. In addition, results will be analyzed according to the on-treatment principle. Participants with documented deviations from the protocol (i.e. false inclusions, participants who did not receive the entire intervention or participants in either the intervention or the control group with incomplete follow-up data) will be excluded from the on-treatment analysis. The results of the ITT analysis will be compared with the results of the on-treatment analysis to assess whether protocol violations have caused bias.

### Economic evaluation

In order to compare the two therapeutic programmes, a cost-effectiveness analysis will be performed. Direct costs will be calculated by adding the costs of the medication, the use of health-related services and the use of pharmaceutical-related services. According to the International Vademecum (Red Book) 2007–2009, the cost of medications will be calculated by determining the price per milligram during the study, including value-added tax, and multiplying it by the daily dose in milligrams and the number of days receiving such treatment. Costs derived from the use of health related services will be calculated considering OBLIKUE unitary cost database [41]. Costs of pharmaceutical related services will be calculated by multiplying the price of an hour of pharmacist attention by the time spent attending patient concerns and needs.

Indirect costs will be calculated considering the days on sick leave and multiplying them by the minimum daily wage in Spain. Finally, total costs will be calculated by adding direct and indirect costs.

Adherence to pharmacological treatment will be used to compare the benefits of each intervention. To determine which of the interventions is best for maximizing benefits, the incremental cost-effectiveness ratio (ICER) will be calculated. The ICER expresses the relation between the costs and effects of one intervention compared with another [42]. To address uncertainty in the ICER sampling distribution, non-parametric bootstrapping will be carried out [43]. Five thousand replications will be carried out for each treatment comparison.

As the duration of the study was only 6 months, neither costs nor outcomes were subject to discounting [42].

## Discussion

The results of this study will provide valuable information for health professionals and policy makers on effectiveness and cost-effectiveness of a pharmaceutical intervention programme in patients with depressive disorders. In the case of proven effectiveness and cost-effectiveness, we would recommend implementing this management intervention into usual healthcare.

Below, design characteristics that involve potential threats to reliability and validity are described.

Firstly, the naturalistic nature of the study and the wide inclusion criteria generate a large inter-subject variability that can reduce the ability to detect differences. On the other hand, that may favour the generalization of the results of this study.

Secondly, two situations may cause contamination bias. Firstly, bias may occur due to the fact that participants of the usual care group share pharmacies with those on the intervention group. To limit this potential contamination bias, interviewers and pharmacists will remind patients not to share information about the appointments with their pharmacists or with other people participating in the study. Secondly, pharmacists participating will receive training in pharmaceutical care in depression which may encourage them to also apply the programme to the control group. In order to limit this bias, pharmacists will be asked to be especially careful not to contaminate the control group with pharmaceutical intervention.

## Competing interests

The authors declare that they have no competing interests.

## Authors' contributions

ASB is the principal investigator and developed the original idea for the research. The study design was done by ASB, PT, MM and MTP. These authors, with the help of MRV and MRF, designed and planned the intervention that is going to be evaluated. ASB and MRV developed the statistical methods. All authors have corrected draft versions and approved the final version of the manuscript.

## Pre-publication history

The pre-publication history for this paper can be accessed here:


